# Flexibility of *Microcystis* Overwintering Strategy in Response to Winter Temperatures

**DOI:** 10.3390/microorganisms9112278

**Published:** 2021-11-01

**Authors:** Pei Cai, Qijia Cai, Feng He, Yuhong Huang, Cuicui Tian, Xingqiang Wu, Chunbo Wang, Bangding Xiao

**Affiliations:** 1 Key Laboratory of Algal Biology, Institute of Hydrobiology, Chinese Academy of Sciences, Wuhan 430072, China; caipei@ihb.ac.cn (P.C.); qjcai@ihb.ac.cn (Q.C.); cctian@ihb.ac.cn (C.T.); xqwu@ihb.ac.cn (X.W.); bdxiao@ihb.ac.cn (B.X.); 2 University of Chinese Academy of Sciences, Beijing 100049, China; 3 Research Academy of Plateau Lake Dianchi, Kunming 671500, China; hefengyunnan@163.com (F.H.); huangyuhongyunnan@163.com (Y.H.)

**Keywords:** *Microcystis*, overwintering, proliferation rate, winter blooms, global warming

## Abstract

*Microcystis* is one of the most common bloom-forming cyanobacteria in freshwater ecosystems throughout the world. However, the underlying life history mechanism and distinct temporal dynamics (inter- and intra-annual) of *Microcystis* populations in different geographical locations and lakes remain unclear but is critical information needed for the development of robust prediction, prevention, and management strategies. Perennial observations indicate that temperature may be the key factor driving differences in the overwintering strategy. This study quantitatively compared the overwintering abilities of *Microcystis aeruginosa* (Ma) in both the water column and sediments under a gradient of overwintering water temperatures (i.e., 4, 8, and 12 °C) using the death and proliferation rates of Ma. The results show that the dynamics of the *Microcystis* overwintering strategy were significantly affected by water temperatures. At 4 and 8 °C, Ma mainly overwintered in sediments and disappeared from the water column after exposure to low temperatures for a long duration, although some *Microcystis* cells can overwinter in the water column for short durations at low temperatures. At 12 °C, most Ma can overwinter in the water column. Rising temperatures promoted the proliferation of pelagic Ma but accelerated the death of benthic Ma. With warmer winter temperatures, pelagic *Microcystis* might become the primary inoculum sources in the spring. Our study highlights the overwintering strategy flexibility in explaining temporal dynamics differences of *Microcystis* among in geographical locations and should be considered in the context of global warming.

## 1. Introduction

Cyanobacterial blooms appear to be increasing in frequency, intensity, and duration due to global warming and anthropogenic activities, and they threaten human and ecosystem health, as well as ecosystem services [1]. *Microcystis* is the most frequently reported bloom-forming cyanobacteria in freshwater ecosystems, and certain *Microcystis* species can produce potent toxins [2,3]. *Microcystis* is ecologically characterized by an annual life cycle comprised of four phenological stages: overwintering, reinvasion, pelagic growth, and sinking out [4,5,6]. In eutrophic lakes, large amounts of *Microcystis* cyanobacterium may sink to the sediment layer during mid-summer and autumn and subsequently survive on the bottom of the lake [7]. The accumulated biomass of *Microcystis* colonies in the sediment could be considered a “seed-bank,” potentially serving as an inoculum source for pelagic blooms after reinvading the water column, thereby contributing to the ecological success and bloom formation of this cyanobacterium in freshwater ecosystems [8,9]. Several bloom mitigation methods have been proposed to eliminate overwintering *Microcystis* populations that are deemed cost-effective [10]. However, the typical four stage life cycle may not in actuality be in accordance with the inter- and intra-annual bloom dynamics of *Microcystis* populations in Chinese lakes.

In China, three large shallow lakes—Lake Taihu, Lake Chaohu, and Lake Dianchi—have suffered from severe *Microcystis* blooms for decades. Previous studies found that the low winter temperatures of Lake Taihu (4.8–7.3 °C) [11] and Lake Chaohu (3.3–6.3 °C) [12] lead to the senescence and cell death of pelagic *Microcystis* but provide optimal conditions for *Microcystis* to overwinter in the sediment [8,13]. However, winter bloom events of *Microcystis* spp. were also observed in Lake Taihu during winters when water temperatures were below 10 °C [14]. *Microcystis* blooms in Lake Dianchi have become a year-round occurrence, with a minimum abundance of 1.3 × 10^7^ cells/L in the winter [15]. It has been clearly demonstrated that *Microcystis* could overwinter, and even bloom, in the water column of Chinese lakes, suggesting that the four-stage life cycle theory does not accurately explain the bloom process of *Microcystis* in these subtropical lakes. In the context of global warming and the expansion of *Microcystis* blooms, overwintering in sediment may not be necessary for *Microcystis* perennial succession, while overwintering, and even blooming, in the water column may be more common. If so, *Microcystis* blooms could become more plaguing and pose an even greater threat to ecosystem health. Therefore, more systematic, and quantitative research on the overwintering patterns of *Microcystis* populations in Chinese lakes and changes in these patterns in response to global climate change are needed.

When overwintering in sediments, the proliferation rate of *Microcystis* can decrease to nearly zero, in which case the death rate is used to determine if the *Microcystis* population density could remain stable. Many researchers have reported that *Microcystis* can survive in sediment for long time periods (>6 years) under low temperatures [16]. Our previous research also revealed that most *Microcystis* spp. remained viable after overwintering at 5 °C in the sediment for over 4 months and could grow after reinvasion of the water column [17]. However, quantitative research concerning the proliferation and death rates of *Microcystis* in the water column at low temperatures are relatively sparse. Yang et al. [18] found that the viability of *Microcystis aeruginosa* showed little decrease under chill-light stress (5 °C, 100 μmol photons m^−2^ s^−1^) within 10 days after preconditioning at 15 °C for 2 days. However, their research mainly focused on the molecular mechanisms involved in the adaptation of *Microcystis* to chill-light conditions. As such, more research is needed to quantify the proliferation and death rates of *Microcystis* overwintering in the water column.

In this study, the growth and survival dynamics of Ma were determined under different culture conditions and overwintering growth capabilities were evaluated. Results yielded possible explanations for the overwintering strategies employed by benthic and pelagic *Microcystis* spp. in three shallow lakes in China and potential ramifications in relation to global warming. In this study, fluorescence staining followed by flow cytometry (FCM) was used to directly quantify the proliferation and death rates of *Microcystis* cells. To quantify the proliferation capacity of *Microcystis* cells, the intracellular protein dye carboxin fluorescein diacetate succinimide ester (CFSE) was used in conjunction with flow cytometry [19,20]. The fluorescent nucleic acid dye, SYTOX Green, was used to quantify damaged cells and indirectly determine the death rate of *Microcystis* cells [21].

## 2. Materials and Methods

### 2.1. Cyanobacteria Cultures

*Microcystis aeruginosa NIES-843* (Ma) was obtained from the Freshwater Algae Culture Collection at the Institute of Hydrobiology, Chinese Academy of Sciences (FACHB-Collection; Wuhan, China). Cells were cultivated in BG11 medium at 25 ± 1 °C and illuminated by white cool fluorescent light lamps (40 μmol photons m^−2^ s^−1^) with a 12:12 h light/dark cycle. The cultures were manually shaken 3 or 4 times each day during incubation. Log-phase cells were centrifuged and resuspended in BG11 medium, and the initial cell density of the experimental cultures was adjusted to 4 × 10^6^ cells/mL.

### 2.2. Experimental Design

*Microcystis* cultures were divided in two, one which was incubated with the fluorochrome CFSE for the detection of cell division, and the other was used for evaluating cell viability and photosynthetic efficiency. The cultures, with or without CFSE, were inoculated into 12-mL sterilized culture tubes containing 10 mL of microalgae cultures. The culture tubes were placed in incubators kept at 4, 8, and 12 °C, respectively. To simulate the luminous environment of the water column and sediment, half of the microalgal cultures were illuminated with 40 μmol photons m^−2^ s^−1^ under a 12:12 h light/dark cycle, serving as the light group, and the other half were covered with tinfoil to serve as the dark group. Microalgal cultures incubated at 25 ± 1 °C and illuminated by white cool fluorescent light lamps (40 μmol photons m^−2^ s^−1^) were used as controls in all experiments. Three tubes of microalgae cultures were randomly sampled weekly from each treatment group. About 1 mL of microalgae cultures with CFSE stain were used for FCM analysis, and 9 mL was returned to the standard growth conditions (25 ± 1 °C, 40 μmol photons m^−2^ s^−1^, under a 12:12 h light/dark cycle) for one week to resume growth.

### 2.3. Flow Cytometric Analysis of Microalgal Cells

Flow cytometric analyses of *Microcystis* cells were performed on a CytoFLEX flow cytometer (Beckman Coulter Inc., Fullerton, CA, USA) equipped with an argon-ion excitation laser (488 nm), forward (FS) and side (SS) light scatter detectors, and four fluorescence detectors corresponding to four different wavelength intervals: 505–550, 550–600, 600–645, and >645 nm. From the start of the experiments, samples were taken weekly from each culture for the different cytometric analyses. Fluorescence of chlorophyll a (>645 nm) was used as a FCM gate to exclude non-microalgal particles. At least 30,000 cells per culture were analyzed.

#### 2.3.1. Cell Division

When incubated with cells, CFSE crosses the cell membrane and binds to the abundant amine groups present in the cytoplasm, after which CFSE acquires identical spectral characteristics to fluorescein after removal of the acetate groups by endogenous intracellular esterases. After each cell division, the cytoplasmic CFSE fluorescence of the mother cell will be distributed equally between daughter cells, allowing analysis of population proliferation at the cellular level [22,23]. The log-phase *Microcystis* culture was stained with fluorochrome CFSE (Sigma-Aldrich, Louis, MO, USA) according the to the methods of Rioboo et al. [20] and Zhou et al. [24]. A CFSE stock solution was prepared by dissolving CFSE powder in DMSO (Sigma-Aldrich) to a final concentration of 10 mM. Next, 130 mL of log-phase *Microcystis* culture (9 × 10^6^ cells/mL) was incubated with 65 μL of CFSE (10 mM) at a final concentration of 5 μM per 6.8 × 10^6^ cells ml^−1^, in BG11 medium for 1 h at 20 °C in the dark. The CFSE-labeled culture was diluted five times with BG11 medium and kept in a culture room for 1 day to allow the cells to recover from the treatments. Cultures without CFSE that cultured under the above conditions were used as controls. Fluorescence of fluorescein was analyzed by the FITC detector (505–550 nm) of the flow cytometer.

#### 2.3.2. Growth Measurement

The cell density of *Microcystis* cultures was measured by FCM. Due to the presence of chlorophyll and phycobiliproteins, *Microcystis* cells showed very strong natural fluorescence [25]. Autofluorescence corresponds to the signal gathered in the PerCP-A detector (660–700 nm), emitted by chlorophyll and related pigments after being excited at 488 nm. This autofluorescence was used to distinguish non-microalgal particles, thus, fluorescence of chlorophyll a (PerCP; 660–700 nm) was used as a gate to select *Microcystis* cells, which showed red fluorescence [26,27].

#### 2.3.3. Cell Viability

The nucleic acid stain SYTOX-Green (Life Technologies, Carlsbad, CA, USA) has been used to distinguish dead and living cells in many studies [28]. Two microliters of SYTOX-Green were added to 1 mL of sample, which was then incubated for 15 min at room temperature in the dark. Fluorescence was analyzed by flow cytometry with excitation and emission wavelengths of 488 nm and 523 nm, respectively. When a cell dies, the integrity of the cell membrane declines, allowing SYTOX Green to easily penetrate cells and bind with DNA to produce a bright green, fluorescent complex which can be analyzed by the FITC detector (505–550 nm) of the flow cytometer [29]. The membrane-damaged cells and integrated membrane cells can be distinguished by differences in fluorescence.

#### 2.3.4. Cell Size, Complexity, and Autofluorescence

The FCM is also equipped with forward (FS) and side (SS) light scatter detectors. Both have been widely applied to measure the cell morphology of phytoplankton [30]. As forward light scatter increases linearly with the square of the cell diameter or cross section, cell size is usually measured by forward scatter [31]. Relating to the refractive index of the cellular content, side light scatter is used to measure intracellular complexity, especially starch content [32]. The samples were analyzed by flow cytometer to identify potential alterations in the cell size or intracellular complexity of *Microcystis* cultures. In the same way, the autofluorescence of *Microcystis* cells (PerCP detector; 660–700 nm) was analyzed by FCM to identify potential changes in the chlorophyll a content of microalgae. Only viable cells were analyzed.

### 2.4. Analyzing the Photochemical Efficiency of Microcystis

The maximum quantum yield for primary photochemistry (Fv/Fm) was measured using a pulse-amplitude-modulated fluorescence monitoring system (Phyto-PAM, Walz, Germany). After a 5-min dark exposure for adaptation, the photosynthetic parameters can be obtained using the PHYTO-PAM analyzer [33].

### 2.5. Calculations and Statistics

The growth rates (week^−1^) of *Microcystis* cultures were calculated using the following formula:

(1) $$\mu \;=\;\frac{\ln\left(N_{t}/N_{0}\right)}{t-t_{0}}$$

where *N_t_* is the cell density (cells/mL) at time *t* (weeks) and *N*_0_ is the cell density at time *t*_0_.

All treatments contained three replicates. All data are presented as mean ± standard errors (SE). Significant differences between treated and control samples were determined by comparing the means. Repeated-measures of multi-factor analysis of variance (MANOVA), followed by least-significant difference (LSD) tests, were applied to determine statistical differences between culture treatments under different temperature and light conditions. Statistical analysis was carried out with the SPSS 26.0 software. Differences were considered to be significant when *p* < 0.05.

## 3. Results

### 3.1. Microcystis Growth under Different Temperature and Light Conditions

**Figure 1:**
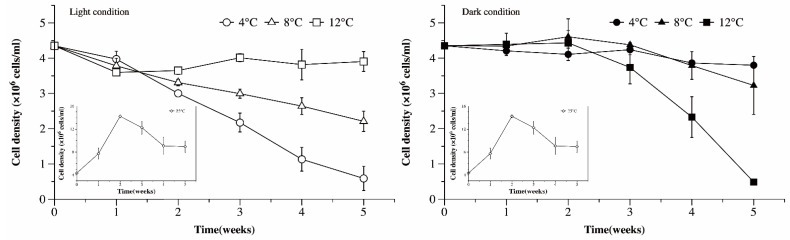
The cell density dynamics of Ma under different temperature and light conditions, as a function of time: Inserts—the control group: The cell density dynamics of Ma under 25 °C and light condition.

The cell density of the Ma cultures kept under control conditions suitable to their proliferation increased sharply from 4.4 × 10^6^ cell/mL to 1.4 × 10^7^ cell/mL in the first two weeks, then gradually decreased to 8.9 × 10^6^ cell/mL over the following three weeks (Figure 1). The light and dark conditions and low temperature used serve as a simplified simulation of the overwintering conditions experienced by Ma cells in the water column and sediment. The cell density of Ma cultures grown at 12 °C in light decreased by about 10% the first week, then experienced little change (*p* > 0.05) afterwards, while the cell density of Ma cultures grown at 4 and 8 °C continued to decline over time during the experiment. After 5 weeks of incubation in light, the cell density of the Ma cultures grown at 4 and 8 °C decreased by 86.5% and 49.3%, respectively, compared with the initial value but only decreased by 10.3% when cultured at 12 °C. Under dark conditions, the cell density of the Ma cultures grown at 4 and 8 °C remained stable during the first three weeks, then significantly declined in the fourth and fifth week, respectively; at 12 °C, the cell density began to decline by the third week. After culturing for 5 weeks under dark conditions at 4, 8, and 12 °C, the survival rates of Ma were 87.3%, 74.2%, and 8.9%, respectively. It can be inferred that increased temperatures were favorable to *Microcystis* overwintering in the water column but unfavorable to *Microcystis* overwintering in sediment.

**Figure 2:**
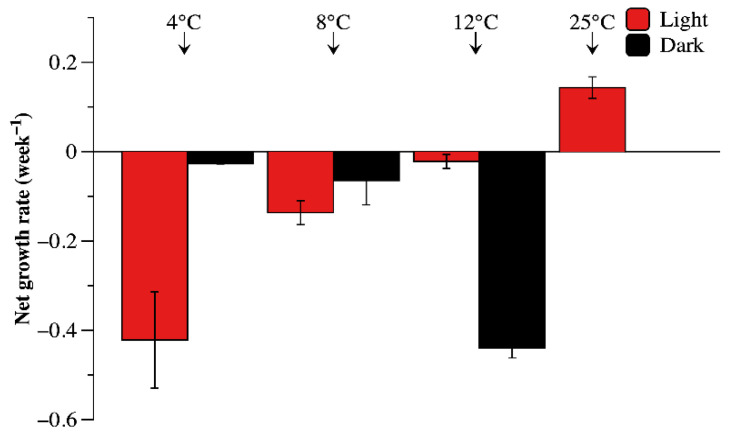
Net Growth rate of Ma cells at different temperature and light conditions.

As shown in Figure 2, the net growth rate of Ma cultures under different temperatures and light conditions were calculated based on the dynamics of Ma cell density at the 5-week incubation period. Low temperature stress had significantly negative effects on the growth rate of Ma. Under light conditions, the net growth rate of Ma cells at 12 °C was close to 0 and significantly higher than that at 4 and 8 °C (*p* < 0.05), indicating the decline in Ma slowed with increasing temperature. On the contrary, increasing temperature accelerated the decline in Ma under dark conditions. The net growth rate of Ma was lower than −0.4 week^−1^ when cultured at both 12 °C in the dark and 4 °C in the light for 5 weeks.

### 3.2. Re-Growth of Microcystis Cells after an Overwintering for a Period

**Figure 3:**
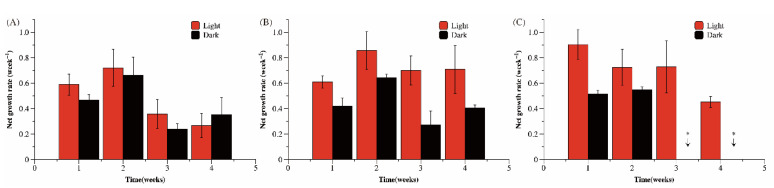
After overwintering for 1–4 weeks under different conditions ((**A**) 4 °C; (**B**) 8 °C; (**C**) 12 °C), Ma cells were cultured at 25 °C in the light. The net growth rate was used to represent the re-growth ability of Ma cells. * Represents a negative growth rate.

When Ma cultures were transferred from low temperature conditions to control conditions (25 °C with illumination) for one week, the re-growth rate was calculated based on changes in Ma cell density during this week (Figure 3). Comparing the re-growth rate of Ma cultures overwintering at 4 °C under light and dark conditions, no significant differences were found (*p* = 0.505). The re-growth rate of Ma cells decreased significantly after overwintering at 4 °C for more than 2 weeks. The length of overwintering time under 8 °C and light conditions had no effect on the re-growth ability (*p* = 0.293). The re-growth rate of Ma overwintering under dark conditions was lower than that under light conditions at 8 °C (*p* = 0.004) or 12 °C (*p* = 0.006). The re-growth rate of Ma cells decreased significantly after overwintering at 12 °C in light for 2 weeks, which did not change with the length of overwintering time. The re-growth rate of Ma was still high after 2 weeks at 12 °C in the dark; however, when the overwintering time exceeded 2 weeks, the re-growth rate was negative, indicating that the ability to re-grow was lost.

### 3.3. Cell Division of Microcystis under Different Temperature and Light Conditions

**Figure 4:**
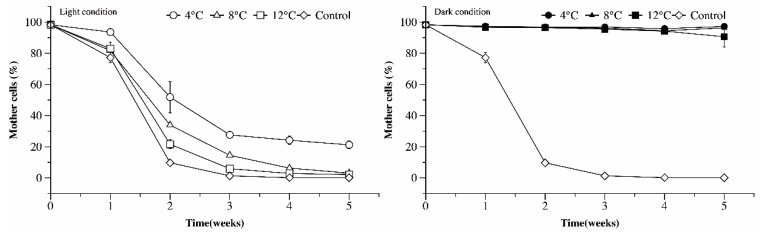
The ratio of mother cells to the sum of mother cells and daughter cells under different temperature and light conditions, as a function of time.

Through fluorescence staining and flow cytometry detection, the densities of undivided (mother) and divided (daughter) cells were quantified (Figure 4). Along with the cell division of Ma cells, the proportion of mother cells decreased. Thus, we use the proportion of mother cells to reveal the proliferation rate of Ma cells.

In the control treatment (25 °C and light), the proportion of mother cells decreased to 10% in the second week and continued to decrease to near zero across the following three weeks. Under low temperature and light conditions, the percentage of mother cells decreased with time during the experiment. At 4 °C in the light, the proportion of mother cells decreased to 30% in the first three weeks and continued to gradually decrease to 21% over the following two weeks. At 8 °C in the light, the proportion of mother cells decreased to 20% over the first three weeks and continued to gradually decrease to 3% over the following two weeks. At 12 °C in the light, the proportion of mother cells decreased to 10% over the first three weeks and continued to gradually decrease to 2% over the following two weeks. Contrary to the results of the light condition treatments, the proportion of mother cells remained stable at 4–12 °C when grown in the dark. These results indicate that even though higher temperatures promoted the proliferation of Ma cells, the cells could continue proliferating even at 4 °C in the light; however, under dark conditions the cells could not proliferate.

### 3.4. Ratio of Undamaged Microcystis Cells under Different Temperature and Light Conditions

**Figure 5:**
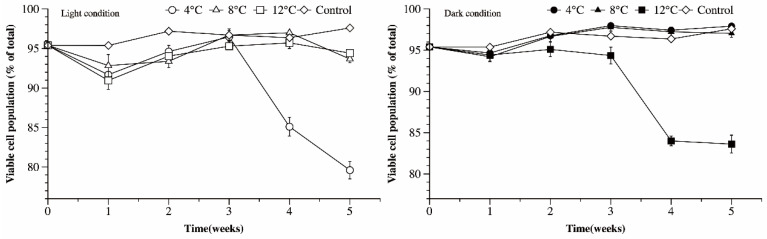
The ratio of undamaged cells to the sum of damaged and undamaged cells at different temperature and light conditions.

Damaged and undamaged cells are distinguished by the fluorescence staining with SYTO and enumerated with flow cytometry. The ratio of undamaged cells to the sum of damaged and undamaged cells was used to represent the variability of overwintering Ma cells. Under light conditions, there was no significant difference in the ratio of undamaged cells grown at 8 and 12 °C (*p* > 0.05). The ratios of undamaged cells grown at 8 and 12 °C both decreased at first, then gradually rose, with the final values being close to the initial value (Figure 5). However, the ratio of undamaged Ma cells in cultures grown at 4 °C decreased significantly after the third week (*p* < 0.05), and 20% of the total population was comprised of damaged cells after 5 weeks. Under dark conditions, Ma exposed to 4 and 8 °C did not lose cell viability, and the undamaged cells accounted for over 97% of the total population after 5 weeks incubation. The ratio of undamaged cells in Ma cultures grown at 12 °C decreased to 84% in the fifth week.

### 3.5. Cell Size and Complexity of Microcystis Cultured under Different Temperature and Light Conditions

**Figure 6:**
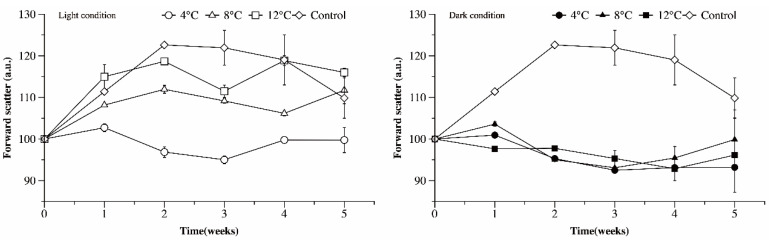
Forward scatter, analyzed by FCM, of Ma cells exposed to different temperature and light conditions as a function of time.

Forward scatter is correlated with the size or volume of a cell or particle. As a unicellular organism, the life cycle of Ma is the same as the cell cycle, which includes three phases: (1) the growth of mother cells, (2) cell division, and (3) the liberation of daughter cells. To maintain cell size, cell volume usually increases during the division process. A down and up trend in the values of forward scatter of Ma cells was observed in both control cultures and those exposed to light conditions, corresponding with the liberation of daughter cells and growth of mother cells in the cell cycle (Figure 6). A significantly higher value of forward scatter in control cultures indicated that more cells were dividing than in cultures exposed to low temperatures. When exposed to darkness, the forward scatter value of Ma cells decreased gradually with time. Under light conditions, the forward scatter value increased with temperature (*p* < 0.05, LSD), while under dark conditions, the temperature had no significant effect on the forward scatter value (*p* = 0.105).

**Figure 7:**
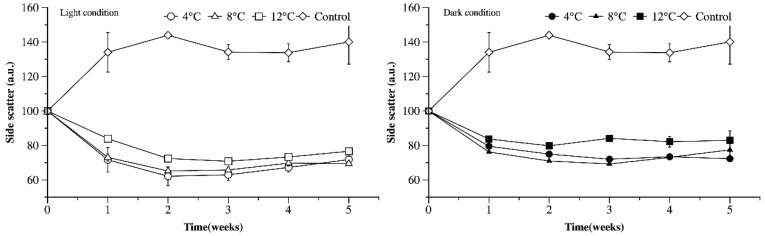
Side scatter, analyzed by FCM, of Ma cells exposed to different temperature and light conditions as a function of time.

Side scatter (SS) is correlated with intracellular complexity. The side scatter values of the Ma cells recorded in control cultures was significantly higher than in the treated cultures (*p* < 0.05). Under light conditions, the value of the side scatter initially declined in the first 2 weeks, then increased gradually (Figure 7). The value of the side scatter of the Ma cells cultured at 12 °C was significantly higher than those cultured at 4 and 8 °C (*p* < 0.05, LSD). A significant decrease in cell side scatter was also observed in the Ma cells exposed to darkness in the first 2 weeks. Subsequently, the side scatter changed little when cultured at 12 °C but continued to decrease slowly at 4 and 8 °C. The side scatter of Ma cells cultured at 12 °C was significantly higher than those at 4 and 8 °C. Since cell division is often characterized by an increase in cell complexity, these results indicate that *Microcystis* cell division likely occurred under light conditions, while the Ma cells did not divide under dark conditions.

### 3.6. Photosynthetic Efficiency of Microcystis Cultures under Different Temperature and Light Conditions

**Figure 8:**
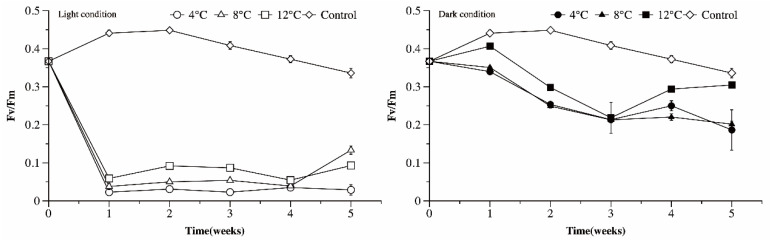
The maximum quantum yield for primary photochemistry (Fv/Fm) of *Microcystis* cultures exposed to different temperature and light conditions as a function of time.

Under light conditions, the Fv/Fm values of the Ma cultures exposed to low temperatures declined dramatically from 0.36 to under 0.1 in the first week (*p* < 0.05). Afterwards, the Fv/Fm values of the Ma cells cultured at 4 °C changed little, while the Fv/Fm values of the Ma cells grown at 8 and 12 °C increased slightly (Figure 8). As the culture temperature increased, so too did the Fv/Fm values of the Ma cultures (*p* < 0.05, LSD). The Fv/Fm values of the Ma cultured in darkness declined over time and were much higher than those cultured in light conditions after 5 weeks of incubation.

## 4. Discussion

### 4.1. Response of Microcystis to Different Temperatures and Light Conditions

By analyzing the net growth rate, proliferation rate (represented by the ratio of divided cells to the sum of divided and undivided cells), and death rate (represented by the ratio of damaged cells to the sum of damaged and undamaged cells), the response of Ma cultures to low temperatures and light/dark conditions was comprehensively evaluated. The net growth rates under all tested conditions (except for the control) were negative, indicating that the biomass of Ma would not increase when temperatures were lower than 12 °C. However, the response of Ma to increasing temperature was different under light and dark conditions. At temperatures ranging from 4 °C to 12 °C in light conditions, rising temperatures increased the proliferation rate and slowed the death rate of Ma cells, thereby increasing the net growth rate of Ma cultures. At 12 °C, the net growth rate was close to zero, showing that the proliferation rate was approximately equal to the death rate. In contrast, under dark conditions, increasing temperature increased the death rate of Ma cells. As Ma cells did not proliferate under dark conditions, the death rate was equal to the net growth rate. This and the results of our previous study [17] indicate that the death rate of *Microcystis* under dark conditions is extremely low, which is beneficial to its long-term survival in sediment during the winter. Field investigations also determined that *Microcystis* can overwinter in sediments under cold and dark conditions and could survive for a long period of time [4,8].

Temperature is one of the dominant environmental factors affecting photosynthetic ability. The Fv/Fm values of Ma cells exposed to low temperature and light conditions fall sharply below 0.1 in the first week, then changed little thereafter. The Fv/Fm values of Ma cells exposed to low temperature and dark conditions, however, decreased slowly and were higher than 0.2 after 5 weeks incubation. This suggests that the photosystem of *Microcystis* under light conditions might be impaired more severely than under dark conditions. Interestingly, when transplanted to normal conditions for re-growth, the re-growth rate of Ma cells overwintering in light conditions were significantly higher than those overwintering in dark conditions. Moreover, the re-growth rate of Ma cells decreased, or ceased entirely, as the length of time of overwintering in dark conditions was extended. These results could suggest that the first decrease in photosynthetic efficiency may be a protective mechanism against photoinhibition occurring under chill and light conditions, rather than permanent photodamage to PSII [34,35], and long exposure to darkness might result in a slow but irreversible deterioration of the physiological state of *Microcystis*.

### 4.2. Overwintering Strategies of Microcystis Populations in Lakes Taihu, Chaohu, and Dianchi

The temporal dynamics of *Microcystis* blooms in Lake Taihu and Chaohu were significantly different from those in Lake Dianchi. In Lake Taihu and Lake Chaohu, *Microcystis* in the water column gradually become dominant when water temperatures increase in spring and summer and gradually disappear when water temperatures decrease in autumn and winter. In contrast, *Microcystis* biomass persisted at high levels throughout the year in Lake Dianchi [15,36,37,38]. Previous studies found that temperature has the most significant effect on the annual duration of blooms [39]. It was also hypothesized that temperature was a key factor in the growth and cessation of planktonic cyanobacteria blooms [40,41].

In this study, we investigated the effects of temperature on the proliferation and death rates of *Microcystis* in the water column and sediment. Our study showed that *Microcystis* biomass decreased significantly over time when cultured at 4 and 8 °C under light conditions, resulting in a higher cell death rate. By fitting the cell density with linear function, the survival times of Ma cells cultured at 4 and 8 °C can be estimated. The results showed that it takes only 5.65 and 10.28 weeks for the cell density of Ma to decrease to zero at 4 and 8 °C, respectively, while the density of Ma remained stable at 12 °C. These results indicate that *Microcystis* can survive in the water column for a short period at low temperatures, and the cell density of *Microcystis* in the water column depends on the duration of exposure to low wintertime temperatures. If the duration of low temperature (4 and 8 °C) exposure exceeded 5.65 and 10.28 weeks, respectively, then *Microcystis* may completely vanish from the water column. Otherwise, there should be a certain number of *Microcystis* that survived. Contrary to that observed in the water column, the overwintering ability of *Microcystis* in sediments is significantly higher at lower temperatures.

Accordingly, in Lake Dianchi, where wintertime water temperatures are higher than 10 °C most of the time, the cell density of pelagic *Microcystis* could remain stable, but the death rate of benthic *Microcystis* would be higher. Moreover, the re-growth ability of benthic *Microcystis* cells after overwintering for several weeks was significantly lower than that of pelagic populations. Thus, most *Microcystis* can overwinter in the water column and become the primary inoculum source in Lake Dianchi. It could be speculated that the high proliferation rate and low death rate of *Microcystis* populations under warmer winter temperatures are important factors driving the persistent winter blooms in Lake Dianchi.

As for Lake Taihu and Lake Chaohu, where winter temperatures are usually below 8 °C, the biomass of pelagic *Microcystis* might gradually decrease because of the high death rates and low proliferation rates. When the duration of low temperatures is short, some *Microcystis* can still survive in the water column and may be able to grow rapidly as spring brings warmer temperatures, thereby contributing to blooms in the summer. When low temperatures are prolonged, the pelagic *Microcystis* cells will disappear from the water column, in which case the death rate of the benthic *Microcystis* populations was very low, creating inoculum in the sediment that feeds subsequent blooms. Regardless of whether winters were warm or cold, surviving *Microcystis* cells may be sufficient to seed the following blooms. This also showed the flexibility of the *Microcystis* overwintering strategy response to different winter temperature conditions.

### 4.3. Response of Winter Microcystis Blooms to Global Warming

Winter temperatures are predicted to be warmer in the future, providing greater opportunities for overwintering pelagic *Microcystis* populations to grow [42]. As winter temperatures increased, the proliferation rate of pelagic *Microcystis* populations also increased while the death rate decreased. In Lakes Chaohu and Taihu, there will likely be more *Microcystis* overwintering in the water column during warmer winters, which might make it easier for pelagic *Microcystis* populations to accumulate to high enough densities to cause more frequent winter *Microcystis* blooms in these lakes. Additionally, with winter temperatures increasing, the overwintering and re-growth abilities of pelagic *Microcystis* are significantly higher than those of benthic *Microcystis*, implying that pelagic *Microcystis* will be the primary sources of inoculum for growth and recruitment in the spring.

## 5. Conclusions

The overwintering strategy of *Microcystis* is related to winter temperature. In shallow lakes with cold winters (e.g., Lake Taihu and Lake Chaohu), *Microcystis* primarily overwinter in sediments. In lakes having warmer water in the winter (e.g., Lake Dianchi), the water column is more suitable for *Microcystis* overwintering. Regardless of whether winter temperatures are warm or cold, the flexibility of the overwintering strategy makes it possible for *Microcystis* to conserve a large enough quantity of cells to seed future blooms. Warmer winter temperatures appear to promote the proliferation of *Microcystis* in the water column, while accelerating its death in the sediment.

## Data Availability

Not applicable.
